# Aflatoxin contamination of groundnut and maize in Zambia: observed and potential concentrations

**DOI:** 10.1111/jam.13448

**Published:** 2017-05-14

**Authors:** P.W. Kachapulula, J. Akello, R. Bandyopadhyay, P.J. Cotty

**Affiliations:** ^1^ School of Plant Sciences University of Arizona Tucson AZ USA; ^2^ School of Agricultural Sciences University of Zambia Lusaka Zambia; ^3^ International Institute of Tropical Agriculture Lusaka Zambia; ^4^ International Institute of Tropical Agriculture Ibadan Nigeria; ^5^ Agricultural Research Service United States Department of Agriculture School of Plant Sciences University of Arizona Tucson AZ USA

**Keywords:** aflatoxin, agroecologies, *Aspergillus* section *Flavi*, groundnut, maize, Zambia

## Abstract

**Aims:**

The aims of the study were to quantify aflatoxins, the potent carcinogens associated with stunting and immune suppression, in maize and groundnut across Zambia's three agroecologies and to determine the vulnerability to aflatoxin increases after purchase.

**Methods and Results:**

Aflatoxin concentrations were determined for 334 maize and groundnut samples from 27 districts using lateral‐flow immunochromatography. Seventeen per cent of crops from markets contained aflatoxin concentrations above allowable levels in Zambia (10 *μ*g kg^−1^). Proportions of crops unsafe for human consumption differed significantly (*P *<* *0·001) among agroecologies with more contamination (38%) in the warmest (Agroecology I) and the least (8%) in cool, wet Agroecology III. Aflatoxin in groundnut (39 *μ*g kg^−1^) and maize (16 *μ*g kg^−1^) differed (*P *=* *0·032). Poor storage (31°C, 100% RH, 1 week) increased aflatoxin in safe crops by over 1000‐fold in both maize and groundnut. The L morphotype of *Aspergillus flavus* was negatively correlated with postharvest increases in groundnut.

**Conclusions:**

Aflatoxins are common in Zambia's food staples with proportions of unsafe crops dependent on agroecology. Fungal community structure influences contamination suggesting Zambia would benefit from biocontrol with atoxigenic *A. flavus*.

**Significance and Impact of the Study:**

Aflatoxin contamination across the three agroecologies of Zambia is detailed and the case for aflatoxin management with atoxigenic biocontrol agents provided. The first method for evaluating the potential for aflatoxin increase after purchase is presented.

## Introduction

Maize and groundnut are preferred crops for both commercial and small‐holder farmers in Zambia. More than 80% of the farmers grow maize for self‐consumption, sale or both in all three agroecologies (Tembo and Sitko 2013) with maize contributing up to 50% of daily calorie intake (FAO 2014). Groundnut, the second most widely cultivated crop, is also grown in all the agroecologies and international demand makes groundnut an important potential source of income. Groundnut and maize are susceptible to aflatoxin contamination. Heavy dependence on these two crops in Zambia may cause significant aflatoxin‐associated health hazards. Liver cancer cases in both Africa and Asia are associated with aflatoxins (Liu *et al*. 2012). Aflatoxin contamination is caused by crop infection by one or more species of aflatoxin‐producing fungi. These fungi disperse from soil, organic matter and alternative hosts to developing crops. Crop infection and subsequent aflatoxin production are high when conditions are hot and dry during crop development and warm and humid after crop maturation and/or harvest (Cotty and Jaime‐Garcia 2007). Consumption of contaminated food may result in cirrhosis, liver cancer, reduced weight gains in livestock, stunted growth and/or immune suppression (Turner *et al*. 2003; Gong *et al*. 2004; Williams *et al*. 2004). Severe acute aflatoxicoses that cause liver necrosis and death have been repeatedly documented in Kenya and India (Lewis *et al*. 2005; Probst *et al*. 2007; Reddy and Raghavender 2007). Enforcement of regulatory limits on aflatoxin concentrations in foods and feeds causes loss of markets for agricultural products and reduced income (van Egmond *et al*. 2007; Wu 2014). Europe and South Africa, with regulatory limits of 4 and 10 *μ*g kg^−1^ total aflatoxin, respectively, are important potential markets for agricultural commodities from Zambia. The country exported over 8000 metric tons of groundnut to Europe in the 1960s. However, this market collapsed due in part to crops found to be unacceptably contaminated in Europe (Sitko *et al*. 2011).

The interplay of climate conditions with cropping systems and fungal community composition influences both the aetiology of contamination and potential remedial measures (Cotty *et al*. 2008; Probst *et al*. 2010). The three agroecologies of Zambia differ in rainfall and temperature (Bunyolo *et al*. 1995). Variation among these agroecologies in aflatoxin incidence is underexplored. Risks posed by communities of aflatoxin‐producing fungi are estimated in part by determining their average aflatoxin‐producing potential (Cotty *et al*. 2008; Probst *et al*. 2010), information that is not available in Zambia. The most effective management strategy for aflatoxin is competitive exclusion of aflatoxin‐producers by atoxigenic genotypes of *Aspergillus flavus* (Cotty and Bayman 1993). Frequencies of atoxigenic fungi may both contribute to explanations of contamination patterns and provide pools of germplasm from which to choose potential biological control fungi.

In order to expand data on aflatoxin incidences in maize and groundnut across agroecologies in Zambia and to identify causal agents of contamination in these regions, aflatoxin concentrations and infecting fungi were determined in crop samples collected from markets in 27 districts across three agroecologies. Weather variables were found to influence contamination and a method to assess the potential for aflatoxin levels to increase in end user hands was developed. Continued safety of foods with low aflatoxins was found dependent on associated fungi and postpurchase storage conditions.

## Materials and methods

### Study area

Zambia lies between 8° and 18° South, and 22° and 34° East of the Greenwich meridian and is divided into three agroecologies (Bunyolo *et al*. 1995). Agroecology III covers northern areas 1100–1700 m above sea level (m a.s.l.) with annual rainfall >1000 mm, and average temperature of 16°C during the growing season (120–150 days between mid‐November and the end of March; Bunyolo *et al*. 1995). Agroecology II extends through central Zambia 900–1300 m a.s.l. receiving between 800 and 1000 mm annual rain, and average temperature of 23–25°C during the growing season (100–140 days between mid‐November and the end of March; Bunyolo *et al*. 1995). Agroecology I includes southern parts of Zambia and valleys below 900 m a.s.l. with <800 mm average annual rainfall and 30°C average temperature during the growing season (80–120 days between mid‐November and the end of March; Bunyolo *et al*. 1995).

### Sampling

**Figure 1:**
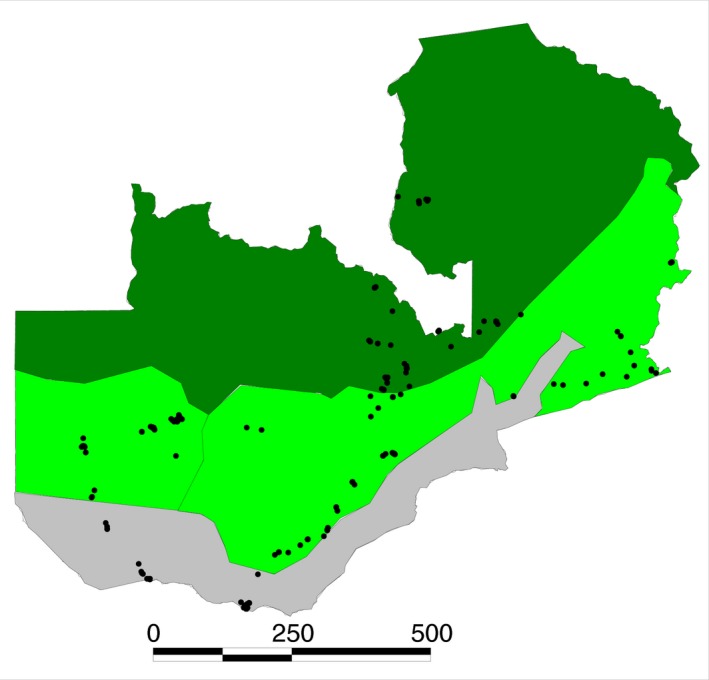
Locations (filled circles) from which crops were collected. Dark green denotes agroecology III, light green denotes agroecology II, and grey denotes agroecology I. Scale bar is kilometers.

In total, 412 maize (250) and groundnut (162) grain samples were obtained from farm storage of subsistence farmers (22) and markets (390) and imported to the USDA, ARS, Laboratory in the School of Plant Sciences, University of Arizona under permit number P526P‐12‐00853 awarded to Peter J. Cotty by the Animal Plant Health Inspection Service of USDA. Samples originated from 27 districts spanning all three agroecologies (Table 1 and Fig. 1). Only samples for which retailers could verify local origin of crops were included. Average temperatures during the growing season and annual rainfall data for the districts in the study were obtained from the Meteorological Department of Zambia (Dr K. Munyinda, personal communication).

**Table 1:** Aflatoxin in maize and groundnut from three agroecologies and 23 districts in Zambia

Agroecology	District	No. of samples	Aflatoxin concentration (*μ*g kg^−1^)		
			Maize	Groundnut	Range
I	Sesheke	32	22^A^	40·5^A^	5·3–621
	Livingstone	11	1·4^B^	5·1^B^	3·9–6·4
	Mean		12^X^	22^X^	
II	Mazabuka	10	107·6^A^	23·4^C^	1·4–5·12
	Nyimba	6	18^B^	NA^b^	ND^a^–101·3
	Kaoma	51	8·4^C^	20·7^C^	3·8–125·1
	Choma	15	5·2^D^	64·7^C^	1–130·3
	Mkushi	3	4·9^D^	NA	3·2–6·5
	Senanga	20	4·8^D^	7^C^	ND–16·4
	Vumbwi	4	3·7^D^	NA	1·8–6·2
	Serenje	4	3·5^D^	NA	1·6–5·2
	Mongu	31	3·3^D^	285·4^B^	ND–3420
	Chadiza	3	2·6^D^	NA	1·7–3·5
	Monze	10	2·4^D^	361·2^A^	1·5–1192
	Kalomo	11	2·3^D^	3·5^C^	1·3–6·2
	Petauke	8	1·8^D^	NA	1·1–2·9
	Kabwe	12	1·6^D^	20·7^C^	1–122
	Kapiri Mposhi	13	1·5^D^	26^C^	1·7–116
	Chipata	17	1·3^D^	NA	1–5·5
	Chibombo	2	3·6	NA	2·4–4·8
	Katete	2	2·6	NA	2·2–3
	Mean		11^X^	90^X^	
III	Mansa	25	60·5^A^	6·7^A^	ND–1416
	Isoka	4	13·8^B^	NA	4·4–40·2
	Mpongwe	5	2·1^B^	6·1^A^	2–2·1
	Mean		25^X(X)^	6^X(X)^	
Overall			16^(X)^	39^(Y)^	

Means followed by the same letter within each agroecology for each crop are not significantly different (*P *<* *0·05) by Tukey‐Kramer's HSD test. Letters x through y (without parenthesis) indicate differences among agroecologies, and between maize and groundnut (in parenthesis) by Tukey‐Kramer's HSD and Wilcoxon's signed‐rank tests respectively.

a ND = below the limit of detection, LOD (LOD = 2 *μ*g kg^−1^).

b NA = not sampled.

### Aflatoxin quantification in ground maize and groundnut

Total aflatoxins were quantified with a GIPSA approved lateral‐flow immunochromatographic assay (Reveal Q+ for Aflatoxin; Neogen Corporation, Lansing, MI) following modifications to the manufacturer's instructions recommended by GIPSA. Each entire crop (maize and groundnut) sample (350–500 g) was ground with a knife mill (Retsch GM200; Retsch GmbH, Haan, Germany) to pass 75% of the ground material through a 20 mesh sieve, mixed thoroughly, and a 50‐g subsample was blended with 250 ml of 65% ethanol and the aflatoxin content determined according to the manufacturer's instructions.

### Fungal isolation and identification

Maize and groundnut samples were weighed, dried to below 8% water content, ground to pass a #12 sieve in a laboratory mill described above and homogenized. Fungi were recovered from ground crop material using dilution plate technique on modified rose Bengal agar (Cotty 1994). Ground crop material (0·1–10 g) was shaken in 50 ml of sterile distilled water for 20 min (100 rev min^−1^) on a reciprocal shaker. Aliquots (100 *μ*l per plate) of the resulting suspension were spread on three plates of modified rose Bengal agar. Plates were incubated (3 days, 31°C, dark) and up to eight colonies of *Aspergillus* section *Flavi* were transferred to 5‐2 agar (5% V8‐juice; 2% agar, pH 5·2) and incubated (7 days, 31°C). Isolations were performed at least twice for each sample. Species and morphotypes were delineated into *A. parasiticus*,* A. flavus* L strain morphotype (average sclerotia diameter >400 *μ*m), and S strain morphotype (average sclerotia diameter <400 *μ*m) (Cotty 1989) using both macroscopic and microscopic characteristics. Fungi with S strain morphology were separated into S_B_ and S_BG_ based on production of either B or both B and G aflatoxins on maize (below).

### Determining potential for aflatoxin formation after market

To determine the potential for aflatoxin concentrations to increase in market maize and groundnut during handling and storage, Simulated Poor Storage Assays (SPSA) were conducted. Uninoculated maize (*n* = 80) and groundnut (*n* = 67, Table 4) market samples with aflatoxin content below 10 *μ*g kg^−1^ were thoroughly hand mixed and 10 g of each was placed onto metal sieves (10 cm diameter) in a sealed plastic box containing a moist sponge (4 cm × 4 cm × 4 cm) and incubated (31°C, 7 days). After incubation, samples were ground in a blender (Waring 7012S; Waring, Torrington, CT) containing 50 ml 70% methanol at high speed for 20 s. The slurry was allowed to settle (5 min) and 4 *μ*l of the supernatant was spotted directly onto thin‐layer chromatography (TLC) plates (Silica gel 60; EMD, Darmstadt, Germany) adjacent to aflatoxin standards (Aflatoxin Mix Kit‐M; Supelco, Bellefonte, PA, USA) containing known quantities of aflatoxins B_1_, B_2_, G_1_ and G_2_. Plates were developed in ethyl ether–methanol–water, 96 : 3 : 1, air‐dried and aflatoxins visualized under 365‐nm UV light. Aflatoxins were quantified directly on TLC plates using a scanning densitometer (TLC Scanner 3; Camag Scientific Inc., Wilmington, NC) running winCATS 1.4.2 (Camag Scientific Inc.).

### Aflatoxin‐producing ability of fungi from purchased crops

Fungal isolates from maize and groundnut were assayed for aflatoxin‐producing potential on sterile maize and groundnut. A randomly selected set of fungi consisting of 54 *A. parasiticus*, 36 S strain morphology fungi and 39 *A. flavus* L strain morphology fungi were inoculated onto undamaged maize and groundnut kernels (10 g in 250 ml Erlenmeyer flask) previously autoclaved for 60 min, cooled to room temperature and moisture adjusted to 30%. Each isolate was cultured (7 days, 100% RH, 31°C) on both maize and groundnut after inoculation with 1 000 000 freshly harvested spores from 7‐day‐old cultures. After incubation, sample cultures were blended in 50 ml of 70% methanol and aflatoxins were quantified with TLC as previously described.

### Data analysis

The total quantity of section *Flavi* fungi from each sample was calculated as colony‐forming unit per gram (CFU per g). Community composition of section *Flavi* was described as percentage of *A. flavus* L strain morphotype (Cotty 1989) undelineated S strain morphotype (Probst *et al*. 2007), and *A*. *parasiticus* recovered from each sample. Quantities of section *Flavi* members were calculated as per cent multiplied by total section *Flavi* CFU per g. Aflatoxin‐producing ability and aflatoxin content were measured in micrograms per kilogram (*μ*g kg^−1^). Means were compared using paired *t*‐test and multiple comparisons were done using analysis of variance general linear models and Tukey's HSD test as implemented in JMP 11.1.1 (SAS Institute, Cary, NC). Association between proportion of crop having >10 *μ*g kg^−1^ with crop type and agroecology were done using chi‐square test of independence as implemented in JMP 11.1.1 (SAS Institute). Relationships between crop aflatoxin concentration with temperature and rainfall in 10 districts were investigated using regression analyses. Associations between aflatoxin increase and fungal proportions were investigated using regression analyses as implemented in JMP 11.1.1 (SAS Institute). Data were tested for normality and, if required, log‐transformed to normalize the distribution before analysis. However, actual means are presented for clarity. All tests were performed at *α *= 0·05. Where transformation did not achieve normality and equal variances, the nonparametric methods, Wilcoxon's rank‐sum and signed‐rank tests were applied.

## Results

### Influences of agroecology and crop host on crop aflatoxin content

The highest average aflatoxin concentration (108 *μ*g kg^−1^) in maize was detected in Mazabuka district while Chipata had the lowest (Table 1). Monze, the district next to Mazabuka, registered the highest average aflatoxin concentration in groundnut (361 *μ*g kg^−1^). On average, there were no significant differences detected (*F*_2,16_ = 0·94, *P *=* *0·40) in maize contamination among agroecologies (Table 1). Similarly, average aflatoxin in groundnuts did not differ significantly (*F*_2,10_ = 1·15, *P *=* *0·36) among agroecologies (Table 1). However, average aflatoxin concentrations were higher by a paired *t*‐test (*t*_13_ = 2·45, *P *=* *0·030) in groundnut (39 *μ*g kg^−1^) than in maize (16 *μ*g kg^−1^) when agroecologies were not considered (Table 1).

Per cent samples exceeding the 4 *μ*g kg^−1^ European regulatory limit for aflatoxin in food was 100 and 73% for groundnut and maize, respectively, in region I, while in region III it was below 30% for both crops (Table 2). The regulatory limits for total aflatoxin in crops intended for human consumption in Zambia is 10 *μ*g kg^−1^. Proportions of maize and groundnut with >10 *μ*g kg^−1^ total aflatoxins were compared in the three agroecologies. The hypotheses that proportion of unsafe crop (i.e. >10 *μ*g kg^−1^) is independent of agroecology and type of crop were tested. There was an association between groundnut safety and agroecology (*P *<* *0·001, Table 3) while none was detected for maize (*P *=* *0·1006, Table 3). The highest proportion of unsafe crop was in region I (58%) while region III had the least (7%). Proportions of unsafe crop depended on crop type (*χ* ^2^_(2, *n* = 291)_ = 15·009, *P *<* *0·001) and unsafe crops were higher in groundnut (25%) than they were in maize (8%, Table 2).

**Table 2:** Aflatoxin distribution by category in agroecologies of Zambia

Agroecology	Total aflatoxin category (*μ*g kg^−1^)	Proportion of samples in category (%)	
		Groundnut	Maize
I	>100	3·8 (1)^a^	0 (0)
	>20	3·8 (1)	20 (3)
	>10	57·7 (15)	20 (3)
	>4	100 (27)	73·3 (11)
	<4	0 (0)	26·7 (4)
II	>100	10·6 (10)	1·1 (1)
	>20	14·9 (14)	3·2 (3)
	>10	21·3 (20)	5·3 (5)
	>4	51 (48)	41·5 (39)
	<4	48·9 (46)	58·5 (55)
III	>100	3·3 (1)	3·1 (1)
	>20	6·7 (2)	9·4 (3)
	>10	6·7 (2)	9·4 (3)
	>4	26·7 (8)	21·9 (7)
	<4	73·3 (22)	78·1 (25)
Overall	>10	25	8

a Values in parentheses refer to number of samples in category.

**Table 3:** Association between proportions of safe groundnut or maize and agroecology

Agroecology	Crop	Aflatoxin safety category		Total
		Safe^a^	Unsafe^a^	
I	Groundnut	b11 (42%)	15 (58%)	26
	Maize	12 (80%)	3 (20%)	15
II	Groundnut	74 (79%)	20 (21%)	94
	Maize	89 (95%)	5 (5%)	94
III	Groundnut	28 (93%)	2 (7%)	30
	Maize	29 (91%)	3 (9%)	32

a Samples below 10 *μ*g kg^−1^ were considered safe, and those above as unsafe (regulatory limit for Zambia).

b Numbers inside and outside parenthesis refer to number of samples and proportion, respectively, in the category. Proportions were compared for each crop using the Freeman–Halton test. *P *<* *0·001 for groundnut and 0·1006 for maize indicate the presence of an association between the proportion of safe groundnut and agroecology, but not maize.

**Figure 2:**
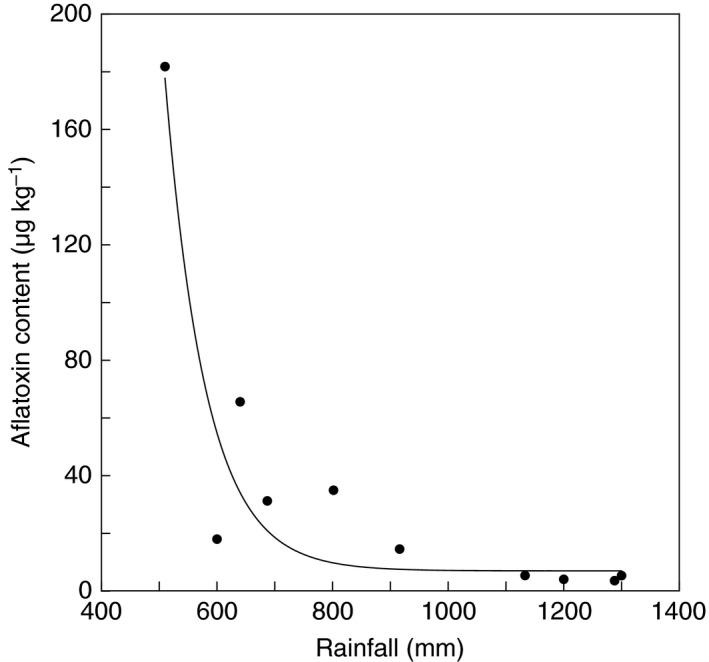
Relationship of crop (maize and groundnut) average aflatoxin content to average annual rainfall in 10 districts of Zambia. Y=10+232911*e^(−0.0141*X)^; R^2^ = 0·89; *P* < 0·001.

**Figure 3:**
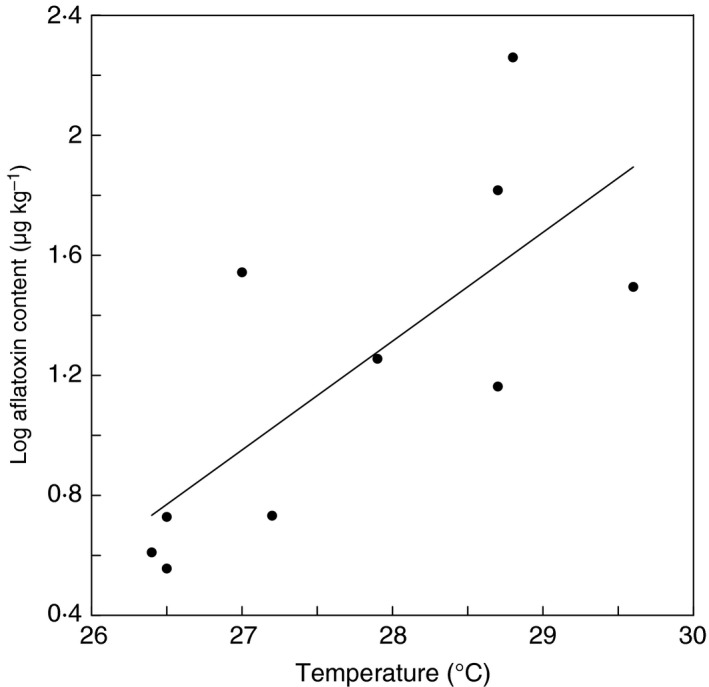
Relationship of log of crop (maize and groundnut) average aflatoxin content to average annual temperature in 10 districts of Zambia. Y = −8·84 + 0·363X; R^2^ = 0·55; *P* < 0·05.

Rainfall significantly (*P *<* *0·001) explained crop aflatoxin content (Fig. 2), whereby increase in rainfall reduced aflatoxins fitting an exponential decay model (*y = *10 + 232 911 × e^(−0·0141 × *x*)^ *R* ^2^ = 0·89). Temperature significantly (*P *<* *0·03) explained crop aflatoxin content (Fig. 3), whereby aflatoxins increased as a function of increase in temperature (*y* = −8·84 + 0·363*x*), *R* ^2^ = 0·55).

### Aflatoxin formation after simulated poor storage

Increases in aflatoxin content of several magnitudes were observed in both maize and groundnut purchased from markets and incubated at 31°C and 100% RH (Table 4). These increases occurred regardless of the agroecology from which the crops originated. In most samples, all four aflatoxins were detected, with total aflatoxin increasing at least 1000‐fold from 3 to 4418 *μ*g kg^−1^ (*t*_34_ = 8·86, *P *<* *0·001) in maize and 30 000‐fold (from 3 to 100 302 *μ*g kg^−1^) in groundnuts (*t*_39_ = 12·19, *P *<* *0·001). Most of the previously safe groundnut (87%) and maize (67%) exhibited toxin increases during incubation at high temperature and high humidity. Although both crops developed lethal levels of aflatoxins (Table 4), the increases were greater in groundnut than maize (*t*_63_ = 3·50, *P *<* *0·001).

**Table 4:** Aflatoxin increases in safe uninoculated incubated maize and groundnuts in SPSA assays^a^

Agroecology	District		Average aflatoxin (*μ*g kg^−1^) in incubated maize		% Maize showing increase	Average aflatoxin (*μ*g kg^−1^) in incubated groundnuts		% groundnut showing increase	Total crop aflatoxin (*μ*g kg^−1^)
			Before^b^	After		Before	After		
I	Sesheke	B_1_	ND^c^	1328		ND	17 593		
		B_2_	ND	29		ND	639		
		G_1_	ND	812		ND	8001		
		G_2_	ND	21		ND	259		
		Total	5·9	2190^a(a)^	50 (*n* = 8)	7·8	26 492^b(b)^	83 (*n* = 6)	28 682^a^
II	Kaoma	B_1_	ND	604		ND	65 298		
		B_2_	ND	41		ND	9710		
		G_1_	ND	682		ND	13 618		
		G_2_	ND	42		ND	3636		
		Total	6·2	1369^(s)(a)^	43 (*n* = 21)	5·3	92 263^(s)(b)^	95 (*n* = 21)	93 632
	Mongu	B_1_	ND	3753		ND	132 384		
		B_2_	ND	131		ND	11 182		
		G_1_	ND	1106		ND	51 536		
		G_2_	ND	59		ND	4440		
		Total	ND	5050^(s)(a)^	76 (*n* = 17)	ND	199,541^(s)(b)^	95 (*n* = 20)	204 591
	Senanga	B_1_	ND	6731		ND	67 617		
		B_2_	ND	276		ND	16 732		
		G_1_	ND	3468		ND	38 904		
		G_2_	ND	129		ND	8202		
		Total	4·8	10 603^(s)(a)^	82 (*n* = 11)	ND	131 455^s(b)^	100 (*n* = 5)	142 058
	Mean			5674^a(a)^		ND	141 086^a(b)^		146 760^b^
III	Mansa	B_1_	ND	1668		ND	25 212		
		B_2_	ND	88		ND	998		
		G1	ND	1053		ND	25 112		
		G2	ND	67		ND	435		
		Total	ND	2876^a(a)^	83 (*n* = 23)	ND	51 758^b(b)^	80 (*n* = 15)	54 634^a^
Average across districts			3^x^	4418^y^		3^(x)^	100 302^(y)^		

a Data are based on aflatoxin produced in uninoculated incubated maize (*n* = 80) and groundnut (*n* = 67) subsamples from safe crops (<10 *μ*g kg^−1^). SPSA = Simulated Poor Storage Assay.

b Before and after columns refer to aflatoxin concentration before and after incubation, respectively.

c ND is none detectable (limit of detection is 2 *μ*g kg^−1^). Aflatoxin chemotypes before incubation not included because quantities were too low to detect.

Letters a, b and c separate means across agroecologies (without parentheses) and between maize and groundnut or in each row (in parentheses). Letters x and y separate means before and after incubation in maize (without parentheses) and groundnut (in parentheses). Means followed by the same letter are not significantly different (*P *<* *0·05) by Wilcoxon's rank‐sum and signed‐rank tests.

### Association of community composition and aflatoxigenicity with increases in crop aflatoxin content after simulated poor storage

The association between community composition and aflatoxin increases under simulated poor storage and toxigenicities of associated fungi was investigated as previously described. Both the per cent (arcsine transformed) and the quantity (log CFU per g) of the *A. flavus* community composed of the L strain morphology fungi inversely explained the per cent increase in crop aflatoxin content in groundnut during incubation (30°C, 100% RH) (for proportion, log *y* = 11·527 615− 5·109 288*x*,* R* ^2 ^= 0·55, *P *<* *0·001; for quantity, log *y* = 11·509575− 1·135883*x*,* R* ^2 ^= 0·34, *P *<* *0·001). The quantity of S strain morphotype explained increases in aflatoxin in incubated groundnut (log *y* = 6·687 114 + 1·0 997 904*x*,* R* ^2 ^= 0·31, *P *=* *0·0015) while that of *A. parasiticus* did not. The total quantity of fungi did not explain aflatoxin increases in incubated groundnut. Aflatoxin increases in incubated maize was not explained by either proportion or quantity of any of the section *Flavi* fungi investigated (Table 5).

**Table 5:** Regression analyses of aflatoxin increase as explained by frequency of members of *Aspergillus* section *Flavi* community^a^

Community component	Intercept	Rate of increase^b^	Coefficient of determination (*R* ^2^)	Model significance (*P*)^c^
Groundnut				
% L^d^	11·527615	−5·109288	0·548	<0·0001
Quantity of L (CFU per g)	9·6943513	−1·135883	0·338	0·0007
% P	8·3508565	2·3851836	0·064	0·1791
Quantity of P (CFU per g)	7·2373955	0·8249238	0·121	0·06
% S	7·6824925	3·3907445	0·0143	0·196
Quantity of S (CFU per g)	6·687114	1·0997904	0·308	0·0015
Total fungi (CFU per g)^e^		−0·047534	0·0001	0·9489
Maize				
% L	11·527615	−1·213759	0·023	0·3819
Quantity of L (CFU per g)	4·5281037	0·3735226	0·04	0·2473
% P	5·9776435	−0·627793	0·001	0·8835
Quantity of P (CFU per g)	5·8385679	0·13752	0·004	0·7224
% S	5·878224	0·7943146	0·004	0·7089
Quantity of S (CFU per g)	5·5567231	0·6588243	0·08	0·0991
Total fungi (CFU per g)		0·4543727	0·053	0·1831

a Data are based on 89 and 67 maize and groundnut samples, respectively, with aflatoxin concentration <10*μ*gkg^−1^.

b This value represents the change in aflatoxin for a unit change in percentage or CFU per g of crop. Negative values reflect aflatoxin reduction.

c Significance set at *P* = 0·05.

d L, P and S represent *A. flavus* L strain morphotype, *A. parasiticus* and S strain morphotype fungi, respectively.

e Total fungi refers to two morphotypes plus *A. parasiticus* combined. Per cent occurrence data were arcsine transformed while CFU per g was log‐transformed prior to analyses.

### Aflatoxin‐producing ability of fungi from purchased crops

Quantification of the relative aflatoxin‐producing potential of 51 *A. flavus* L strain morphotype (33 isolated from maize and 18 from groundnut), 54 *A. parasiticus* (28 isolated from maize and 26 from groundnut) and 38 S strain morphotype fungi (16 isolated from maize and 22 from groundnut) obtained from samples used in the incubation experiments was done on both maize and groundnut as previously described. Ten (three from maize and seven from groundnut) of the S strain morphotype fungi produced only B aflatoxins (thus designated S_B_) and 28 (13 from maize and 15 from groundnut) produced both B and G aflatoxins (thus designated S_BG_) (Table 6). There were significant differences in aflatoxin B_1_ (*F*_3,139_ = 41·50, *P *<* *0·001) and total aflatoxin (*F*_3,139_ = 51·55, *P *<* *0·001) production among the section *Flavi* members. On groundnut the average total aflatoxin produced by isolates of *A. parasiticus* (237 000 *μ*g kg^−1^) was significantly higher (*P *<* *0·0126) than that produced by G aflatoxin‐producing S strain morphotype fungi (91 455 *μ*g kg^−1^) by Student's *t*‐test. Quantities of aflatoxins produced on groundnut by S strain morphotype fungi that produced only B aflatoxins (4157 *μ*g kg^−1^) did not differ significantly (*P *=* *0·139) from that produced by *A. flavus* L strain morphotype isolates (4168 *μ*g kg^−1^); although each produced significantly less aflatoxins than either *A. parasiticus* (*P *<* *0·001 for S_B_ and for *A. flavus* L strain morphology) and S strain morphotype fungi that produced both B and G aflatoxins (*P* = 0·0051 and *P *<* *0·001 for S_B_ and *A. flavus* L strain morphotype respectively) by Student's *t*‐test. Unlike on groundnut, the total aflatoxin produced by S_BG_ (265 748 *μ*g kg^−1^) and *A. parasiticus* (192 398 *μ*g kg^−1^) on maize did not differ significantly (*P *=* *0·5187, Student's *t*‐test), but both taxa produced significantly more aflatoxins than the other taxa (Table 6). Aflatoxin production by *A. parasiticus* did not significantly differ between maize and groundnut (*t*_53_ = 0·14, *P *=* *0·8912) by paired *t*‐test. However, significantly greater quantities of aflatoxins were produced on maize than groundnuts by both fungi with S strain morphology and the *A. flavus* L strain morphotype (*P *<* *0·001; Table 6). Fungi produced comparable amounts of aflatoxin irrespective of crop of origin (Table 7).

**Table 6:** Mean toxin‐producing abilities of section *Flavi* fungi isolated from crops containing <10 *μ*g kg^−1^ prior to incubation

Taxon^a^	No. of isolates^b^		Average aflatoxin (*μ*g kg^−1^)				Crop average (*μ*g kg^−1^)
			Maize	Range	Groundnut	Range	
P	33, 18	B_1_	80 104^a(x)^	5527–219 006	136 098^a(x)^	2166–3 048 587	108 101^a^
		B_2_	2232	ND–14 485	2317	ND–9042	
		G_1_	106 917	123–761 700	96 397	349–356 995	
		G_2_	3146	ND–29 233	2272	ND–13 046	
		Total	192 398^(a)(x)^	7408–497 384	237 085^(a)(x)^	2642–3 188 272	214 742^(a)^
S_BG_ c	13,15	B_1_	118 583^a(x)^	146–1 038 204	32 399^b(y)^	125–251 500	75 491^ab^
		B_2_	1759	ND–9715	547	ND–5599	
		G_1_	143 685	ND–1 415 343	57 252	ND–613 519	
		G_2_	1721	ND–11 398	1257	ND–19 021	
		Total	265 748^(a)(x)^	248–2 453 547	91 455^(b)(y)^	125–814 764	178 602^(a)^
S_B_ c	3,7	B_1_	40 780^b(x)^	ND–120 298	4008^c(y)^	ND–14 069	22 394^bc^
		B_2_	2017	ND‐4409	149	ND–932	
		G_1_	ND^d^	ND	ND	ND	
		G_2_	ND	ND	ND	ND	
		Total	42 798^(b)(x)^	ND–124 489	4157^(c)(y)^	ND–15 001	23 477^(b)^
L	26,18	B_1_	12 888^c(x)^	ND–153 433	4011^c(y)^	ND–58 392	8450^c^
		B_2_	838	ND–10 850	157	ND–3067	
		G_1_	ND	ND	ND	ND	
		G_2_	ND	ND	ND	ND	
		Total	13 727^(c)(x)^	ND–164 283	4168^(c)(y)^	ND–61 460	8948^(b)^

a Taxon consists of P (*A. parasiticus*), L (*A. flavus* L strain morphotype) and S strain morphotype.

b Number before and after the comma represents number of isolates that originated from maize and groundnut respectively.

c *Aspergillus* section *Flavi* fungi with S strain morphology in Southern Africa may be either the S strain morphotype of *A. flavus*,* A. minisclerotigenes*, the unnamed taxon S_BG_ from West Africa or the fungus associated with lethal Aflatoxicosis in Kenya.

d ‘ND’ means below the limit of detection, LOD (LOD = 20 *μ*g kg^−1^).

Letters a, b and c separate means in each column for aflatoxin B_1_ (without parenthesis) and total aflatoxin (in parenthesis) among P (*A. parasiticus*), L (*A. flavus* L strain morphotype) and S strain morphotype. The letters x and y separate means in each row. Means followed by the same letter are not significantly different (*P *<* *0·05) by Tukey–Kramer's HSD for between morphotype comparison and Student's *t*‐test for within morphotype comparison.

**Table 7:** Comparing aflatoxigenicity of *Aspergillus* section *Flavi* isolates from maize and groundnuts

Morpho‐group	Originating substrate	No. of isolates	Aflatoxin on maize		Aflatoxin on groundnuts	
			B_1_	Total	B_1_	Total
P	Maize	28	93 440^a(x)^	212 659^ax^	189 852^a(x)^	312 464^ax^
	Groundnut	26	65 742^b(x)^	170 579^bx^	78 210^a(x)^	155 908^ax^
S_BG_	Maize	13	122 539^f(x)^	278 374^fx^	19 774^f(y)^	74 027^fy^
	Groundnut	15	115 155^f(x)^	254 806^fx^	43 341^f(y)^	106 560^fy^
S_B_	Maize	2	42 234^j(x)^	45 054^jx^	8298^j(x)^	8764^jx^
	Groundnut	6	53 889^j(x)^	56 311^jx^	3914^j(y)^	4007^jy^
L	Maize	26	7211^q(x)^	7510^qx^	2716^q(y)^	2804^qy^
	Groundnut	13	36 143^q(x)^	38 834^qx^	10 305^q(y)^	10 746^qy^

Letters a/b, f/g j/k and q/r separate means from the two crops within each morpho‐group in the column while the letters x and y compare B_1_ (in parenthesis) and total aflatoxin (without parentheses) within each row. Means followed by the same letter are not significantly different (*P *<* *0·05) by Student's paired *t*‐test (within each row) or Student's *t*‐test (within each morpho‐group in each column).

## Discussion

To determine the extent of the problem attributable to aflatoxin contamination of food, both detected concentrations and consumption habits must be taken into consideration (Marasas 1997). In Zambia, the majority of the population consumes maize daily with on average 50% of calories derived from maize‐based food (FAO 2014). Groundnuts are an important source of energy in sauces and vegetables and as a snack and are both produced and consumed across the nation. Thus, unacceptable aflatoxin contents in 17% of these primary staple crops from markets, as found in the current study, provides a greater risk to the population compared to regions with higher incidences and concentrations but with reduced rates of consumption and diets that are more diverse. In the current study, sufficient frequencies and concentrations of aflatoxins were detected to support development of aflatoxins management strategies for Zambia based on health concerns and not just the well‐established impact of aflatoxins on access to international markets. Successful management strategies developed for Zambia will have to take into account the very high average aflatoxin‐producing potentials of the fungal communities detected in the current study (Table 6).

### Influences of agroecology on aflatoxin concentration

Environmental events such as drought, temperature extremes, or rain on the mature crop have large impacts on crop aflatoxin content (Cotty and Jaime‐Garcia 2007). In a similar manner, perennial contamination is often characteristic of production areas with environmental conditions that favour both reproduction of the causative fungi and infection of susceptible crops. Contamination was most frequent and severe in the warmest production areas of Zambia (Fig. 3). Aflatoxin is widely distributed in maize and groundnut produced in Zambia (Table 1). Unsafe levels of aflatoxins occurred in all three agroecologies with average concentrations above the legal limit of 10 *μ*g kg^−1^ in all agroecologies for maize (Table 1) and agroecologies I and II for groundnut (Table 1). Aflatoxin levels do not differ significantly among agroecologies (Tables 1 and 2; Kankolongo *et al*. 2009). However, the frequency of unsafe groundnut (>10 *μ*g kg^−1^) depended on agroecology (Table 3). The results for groundnut are consistent with climate being an important factor dictating the extent of contamination with the highest proportions of unsafe groundnut in agroecology I (warm and dry) and the lowest agroecology III (wetter and cooler).

The primary climatic differences among the agroecologies in Zambia are temperature and rainfall. Levels of aflatoxin were influenced by rainfall (Fig. 2) and temperature (Fig. 3). Aflatoxins increased when temperature increased, and decreased with higher annual quantity of rain resulting in the highest frequencies of unsafe crops in the warmest, driest regions. Low moisture combined with high temperature results in highly stressed plants with increased susceptibility to invasion by aflatoxin‐producing fungi (Cotty *et al*. 1994, 2008). Warm regions favour growth of aflatoxin‐producing fungi (Cotty *et al*. 1994, 2008) and stressed plants expend more energy maintaining crop development and less on defence activities such as phytoalexin production (Wotton and Strange 1987). Hot dry conditions cause reduced tissue integrity in developing plants (Odvody *et al*. 1997) and trigger early onset of developmental processes such as flowering (Doster and Michailides 1995; Hadavi 2005), which creates entry points that allow infection by aflatoxin‐producing fungi. However, rainfall and temperature alone do not adequately explain the observed variation in aflatoxin levels. For example, although Sesheke and Livingstone districts fall in the same agroecology and have comparable temperatures and rainfall, the two districts differed in aflatoxin levels in both maize and groundnut (Table 1).

### Exposure to aflatoxins through consumption of maize and groundnut

Maize and groundnuts are both important food security crops in Zambia (Sitko *et al*. 2011; Tembo and Sitko 2013). In the current study, groundnut had both higher average aflatoxin concentrations and a greater frequency of contamination than maize (Tables 1 and 2). However, maize is consumed in higher quantities and at higher frequencies than groundnut, providing up to 50% of daily calorie intake (FAO 2014). As such, aflatoxin levels in maize, even though lower in concentration, pose a greater potential health burden than groundnut contamination. Average aflatoxin concentrations in maize are lower than those frequently reported in Kenya and much lower than those causing lethal acute aflatoxicoses in India and Kenya (Lewis *et al*. 2005; Reddy and Raghavender 2007). However, a portion of the maize crop in Sesheke, Monze, Mongu and Mazabuka districts had aflatoxin concentrations sufficient to result in acute lethal aflatoxicosis if those crops served as the primary source of calories (Table 1). In the current study, crops were examined over both more diverse environments and greater expanses of Zambia than previously (Kannaiyan *et al*. 1987; Kankolongo *et al*. 2009; Mukanga *et al*. 2010; Bumbangi *et al*. 2016) and greater quantities of aflatoxins were detected. These observations indicate a need for interventions to reduce aflatoxins, particularly in the warmer drier regions, where poor crop storage, common among small‐scale farmers, may exacerbate contamination (Kankolongo *et al*. 2009).

### Influences of fungal community structure on potential for crop contamination after market

The quantities of aflatoxins both at harvest and at markets may not fully represent the risk of aflatoxin exposure from the crop because crop‐associated fungal communities remain with crops until consumption and may produce aflatoxins during handling, storage and processing (Cotty *et al*. 1994, 2008). Fungal communities on crops from each of Zambia's agroecologies have high average aflatoxin‐producing potentials (Table 6). Aflatoxins increase in poorly stored crops after harvest (Cotty *et al*. 1994, 2008; Jaime *et al*. 2013). Biocontrol fungi retained on crops after harvest reduce aflatoxin increases in storage (Atehnkeng *et al*. 2014). However, risks of aflatoxin increases attributable to crop‐associated fungi after harvest previously have been difficult to quantify. Relative risk of aflatoxin increases from crop‐associated fungi was quantified in the current study with an SPSA. Risk quantified by SPSA varied among crops from 4418 to 100 302 *μ*g kg^−1^ (Table 4), with increases higher in groundnuts than maize. These aflatoxin risks, and mitigation options, need to be understood by farmers, processors and end users. Some crops expressed no risk of increase in the SPSA assay (Table 4), possibly indicating fungal communities inadequate to support contamination (Cotty *et al*. 2008; Probst *et al*. 2010). Presence of atoxigenic *A. flavus* in fungal communities can prevent postharvest aflatoxin increases (Atehnkeng *et al*. 2016).

*Aspergillus* section *Flavi* communities from crops subjected to SPSA consisted of the *A. flavus* L strain morphotype, *A. parasiticus* and fungi with S strain morphology that produced either only B aflatoxins (S_B_) or both B and G aflatoxins. Crops with high frequencies of the L strain morphotype prior to incubation had little or no aflatoxins form during SPSA (Table 5). Most *A. flavus* L strain morphotypes from Zambia were capable of producing little or no aflatoxins (Table 6). Thus, the results from SPSA are similar to results from field trials where atoxigenic *A. flavus* biocontrol agents reduce crop aflatoxin content both prior to and after harvest (Atehnkeng *et al*. 2014, 2016). During SPSA groundnut aflatoxin content increases were greatest when high incidences of either S strain morphotype fungi or *A. parasiticus* were present (Table 5). Both S strain morphotype fungi and *A. parasiticus* consistently produce high concentrations of aflatoxins (Cotty and Cardwell 1999; Jaime‐Garcia and Cotty 2006; Cotty *et al*. 2008; Probst *et al*. 2010).

Aflatoxin increases in SPSA were higher in groundnut than in maize (Table 4), even though these crops originated from the same areas. However, more aflatoxins formed in maize inoculated with either S_BG_, S_B_ or *A. flavus* fungi than groundnut (Table 6). The two crops became similarly contaminated when inoculated with *A. parasiticus*. Fungi isolated from maize were just as toxigenic as those originating from groundnut (Table 7). Differential performance of the two crops in SPSA is therefore not attributable to peanut supporting greater aflatoxin production or containing isolates more toxigenic than maize. This reinforces the above observations that risk of aflatoxin contamination during SPSA, and presumably in the hands of the consumer, is most related to the mix of fungi on the crop. Associations between community composition and aflatoxin increases in the current study may be applied to aflatoxin management in Zambia. By modifying fungal community composition to increase proportions of atoxigenic L strain morphotype fungi in the field and eventually on the crop, we could achieve protection not only prior to harvest but also in storage (Atehnkeng *et al*. 2014, 2016).

Aflatoxin contamination of maize and groundnut is common in Zambia and crops purchases with low aflatoxin content are frequently associated with fungi that may form aflatoxins in crops during handling and storage. Aflatoxins occurred in all agroecologies of Zambia with the highest contamination in warm, dry regions. A method for quantifying relative risk of crops to increases in aflatoxin content under poor storage was developed. The assay might be refined by simulating the range of conditions occurring during on‐farm storage in regions of concern. Compositions of fungal communities associated with crops prestorage dictated aflatoxin increases in storage with crops naturally containing atoxigenic *A. flavus* experiencing smaller increases. Consumers may purchase and keep groundnut and maize for long periods increasing vulnerability to aflatoxin increases. Modifying compositions of fungal communities associated with crops prior to harvest with biological control technology should reduce aflatoxin contamination incidences in warm dry agroecologies and reduce increases when proper handling and storage conditions are not practiced (Atehnkeng *et al*. 2014; Bandyopadhyay *et al*. 2016).

## Conflict of Interest

The authors have no conflict of interest to declare.
